# Methylation of the Phospholipase A2 Receptor 1 Promoter Region in Childhood B Cell Acute Lymphoblastic Leukaemia

**DOI:** 10.1038/s41598-020-65825-0

**Published:** 2020-06-03

**Authors:** Markus Friedemann, Katharina Gutewort, Dana Thiem, Brit Nacke, Carsten Jandeck, Björn Sönke Lange, Olga Sukocheva, Meinolf Suttorp, Mario Menschikowski

**Affiliations:** 1 Institute of Clinical Chemistry and Laboratory Medicine, University Hospital Carl Gustav Carus, Technical University of Dresden, 01307 Dresden, Germany; 2 Department of Paediatrics, University Hospital Carl Gustav Carus, Technical University of Dresden, 01307 Dresden, Germany; 3 School of Health Sciences, Flinders University of South Australia, Bedford Park, 5042 Australia; 4 Medical Faculty, Paediatric Haemato-Oncology, Technical University, 01307 Dresden, Germany

**Keywords:** Tumour biomarkers, Haematological cancer

## Abstract

Acute lymphoblastic leukaemia (ALL) is the most common form of paediatric cancer and epigenetic aberrations are determinants of leukaemogenesis. The aim of this study was to investigate the methylation degree of a distinct phospholipase A2 receptor 1 (PLA2R1) promoter region in paediatric ALL patients and to evaluate its relevance as new biomarker for monitoring treatment response and burden of residual disease. The impact of PLA2R1 re-expression on proliferative parameters was assessed *in vitro* in Jurkat cells with *PLA2R1* naturally silenced by DNA methylation. Genomic DNA was isolated from bone marrow (BM) and peripheral blood (PB) of 44 paediatric ALL patients. *PLA2R1* methylation was analysed using digital PCR and compared to 20 healthy controls. Transfected Jurkat cells were investigated using cell growth curve analysis and flow cytometry. *PLA2R1* was found hypermethylated in BM and PB from pre-B and common ALL patients, and in patients with the disease relapse. *PLA2R1* methylation decreased along with leukaemic blast cell reduction during ALL induction treatment. *In vitro* analysis revealed an anti-proliferative phenotype associated with PLA2R1 re-expression, suggesting a tumour-suppressive function of PLA2R1. Collected data indicates that *PLA2R1* promoter methylation quantitation can be used as biomarker for ALL induction treatment control, risk stratification, and early detection of ALL relapse.

## Introduction

Acute Lymphoblastic Leukaemia (ALL) is the most frequent form of childhood cancer worldwide^1,2^. More than 7000 new cases of ALL were diagnosed in the European Union in 2018, with a peak incidence between two and five years of age^3,4^. ALL is characterized by an altered proliferation of lymphoid progenitor cells and a subsequent accumulation of leukaemic blast cells in the bone marrow, blood, and other organs^5–7^. Primary ALL diagnosis is based on the microscopic analysis of bone marrow aspirates^7^. Subtypes of childhood ALL are defined by immunohistological identification of the affected cell lineage (B- or T-cell), chromosomal and genetic alterations, and the cell differentiation status^8^. In addition to germline and somatic mutations, epigenetic alterations (especially DNA promoter hypermethylation) contribute to ALL subtype classification^9^ and may have prognostic value as an indicator of the likelihood of relapse under standardized treatment^10–12^.

The phospholipase A2 receptor 1 (PLA2R1) is a type I transmembrane receptor^13^ that is suggested to play a crucial role in the regulation of cellular senescence/apoptosis in primary human fibroblasts^14^ and different breast cancer cell lines^15–18^. Our previous study was the first to establish that epigenetic mechanisms are involved in the regulation of PLA2R1 expression in leukaemic cells^19^. According to the *PLA2R1* bisulfite sequencing analysis, 77 CpG sites at −473 bp to +586 bp from exon 1 were found to be hypermethylated in blood leukocytes of adult patients with acute myeloid leukaemia compared to healthy individuals. *PLA2R1* methylation quantification by methylation-sensitive high-resolution melting analysis demonstrated a significantly higher methylation degree in adult leukaemia patients. Additionally, the *PLA2R1* methylation degree was found to increase with disease stage progression in a group of myelodysplastic syndrome (MDS) patients^19^. The analysed values correlated with the International Prognostic Scoring System (IPSS) classification, suggesting that *PLA2R1* methylation measurement can be used as an additional biomarker for risk stratification. Preliminary analysis of the *PLA2R1* methylation degrees of high-risk MDS and AML patients during azacitidine treatment indicated that the response to treatment also correlated with the *PLA2R1* methylation degrees, and measuring quantitatively the receptor methylation was considered a useful early indicator for the requirement of follow-up therapy^19^. Furthermore, our study provided evidence that *PLA2R1* promoter methylation is inversely correlated with PLA2R1 expression in the human T lymphocyte acute leukaemia (Jurkat) cell line^19^, which is extensively used to investigate ALL^20–22^.

Based on these previous findings, the aim of the present study was to investigate the following: (i) whether the *PLA2R1* promoter is also hypermethylated in patients with childhood ALL at diagnosis in comparison to healthy individuals; (ii) whether the *PLA2R1* promoter methylation in blood leukocyte DNA can be used as a biomarker for treatment response and control of residual disease. Additionally, the effect of PLA2R1 expression on cell proliferation and apoptosis/necrosis of Jurkat cells as a cell model for childhood ALL was assessed.

## Results

### Differential *PLA2R1* promoter methylation in healthy and childhood ALL samples at diagnosis

**Figure 1:**
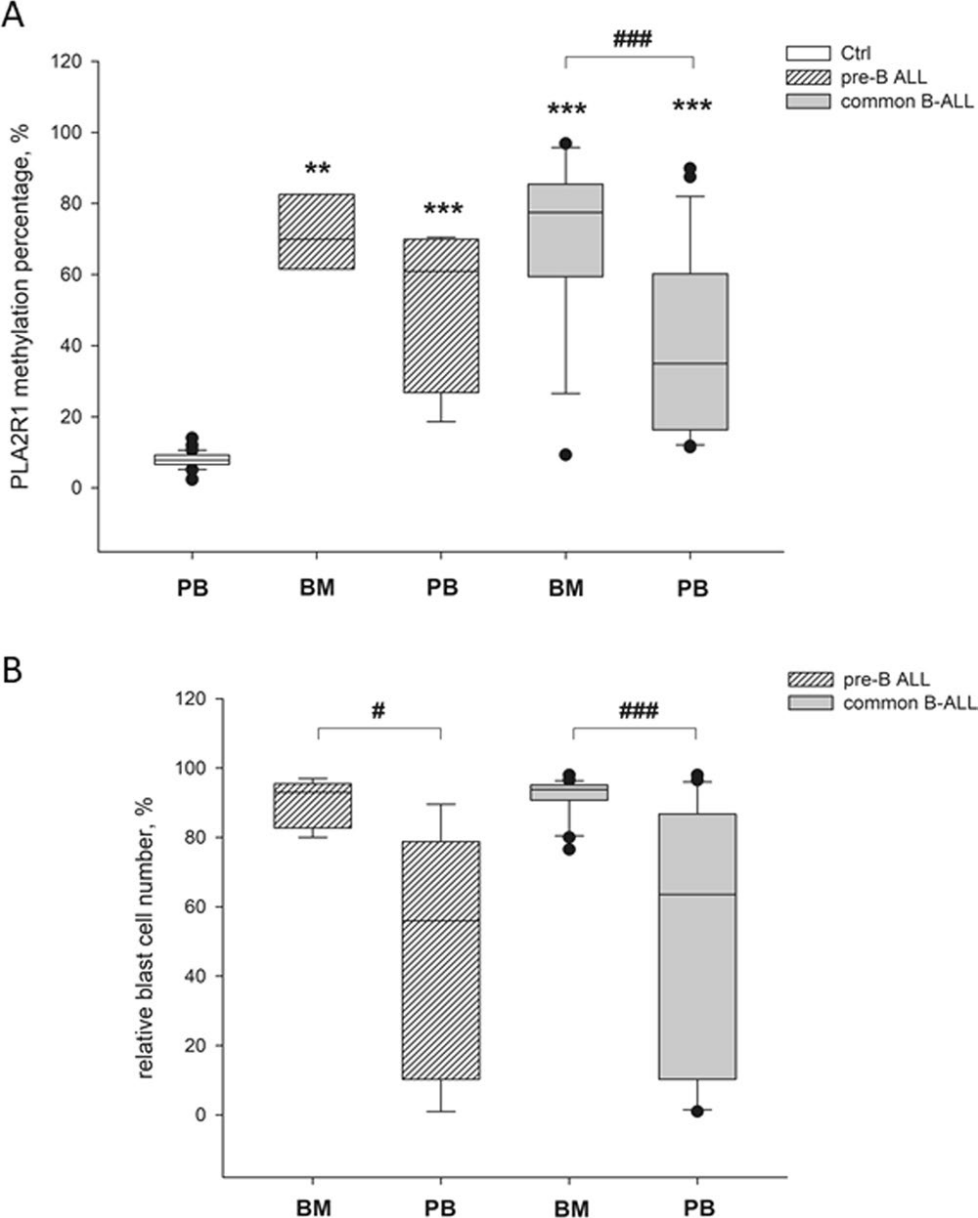
Differential *PLA2R1* promoter methylation and blast cell occurrence in healthy and childhood ALL samples. Box plots consist of the median as ‘center value’, the 25^th^ and 75^th^ percentiles as box edges, and the 10th and 90th percentiles as whisker boundaries. (**A**) Percentage of *PLA2R1* promoter methylation at diagnosis was determined in PB from healthy children (Ctrl, n = 20) and in BM aspirates or PB from children with pre-B cell (n_BM_ = 3, n_PB_ = 5) or common ALL (n_BM_ = 17, n_PB_ = 19) using droplet digital PCR. (**B**) The relative blast cell number (number of blast cells in relation to the number of total leukocytes in %) in BM aspirates and PB of childhood pre-B cell (n_BM_ = 5, n_PB_ = 5) or common ALL samples (n_BM_ = 21, n_PB_ = 22) were determined at diagnosis using light microscopic and flow cytometric analysis. The symbols * and # indicate significant differences compared to the healthy control group or between marked cohorts, respectively. Levels of significance are defined as p < 0.05 (#), p < 0.01 (**), and p < 0.001 (***, ###).

To investigate the effect of PLA2R1 in childhood ALL, the *PLA2R1* promoter methylation status was analysed by droplet digital polymerase chain reaction (ddPCR) in PB samples and BM aspirates of children with ALL and AML. The samples were then compared to a healthy, age-matched control group (Fig. 1).

The mean *PLA2R1* promoter methylation percentage of the healthy, age-matched control group was 7.8% ± 2.3%. The 97.5% percentile of the control group was estimated 12.05% and was defined as cutoff. In comparison to the control group, *PLA2R1* methylation was approximately nine times higher in the BM of patients at diagnosis of pre-B (71.3% ± 8.6%, p = 0.005) and common ALL (70.7% ± 22.1%, p < 0.001) (Fig. 1A). In PB samples, the mean *PLA2R1* methylation percentage was 6.5 or 5.1 times higher in pre-B (50.9% ± 20.6%, p < 0.001) and common ALL samples (40.2% ± 25.7%, p < 0.001) in comparison with the control group, respectively (Fig. 1A). In the common ALL cohort, significant differences between *PLA2R1* methylation of BM and PB samples were observed (p < 0.001).

The mean relative blast cell numbers of BM from pre-B and common ALL patients were 89.9% ± 6.2% and 91.7% ± 5.8%, respectively (Fig. 1B). Thus, in BM aspirates at diagnosis the relative leukaemic blast cell number was generally higher than 76%. However, the *PLA2R1* promoter methylation levels varied between 59–97%, excluding one patient who was tested with 82% blasts at a slightly lower *PLA2R1* promoter methylation of 31%.

**Figure 2:**
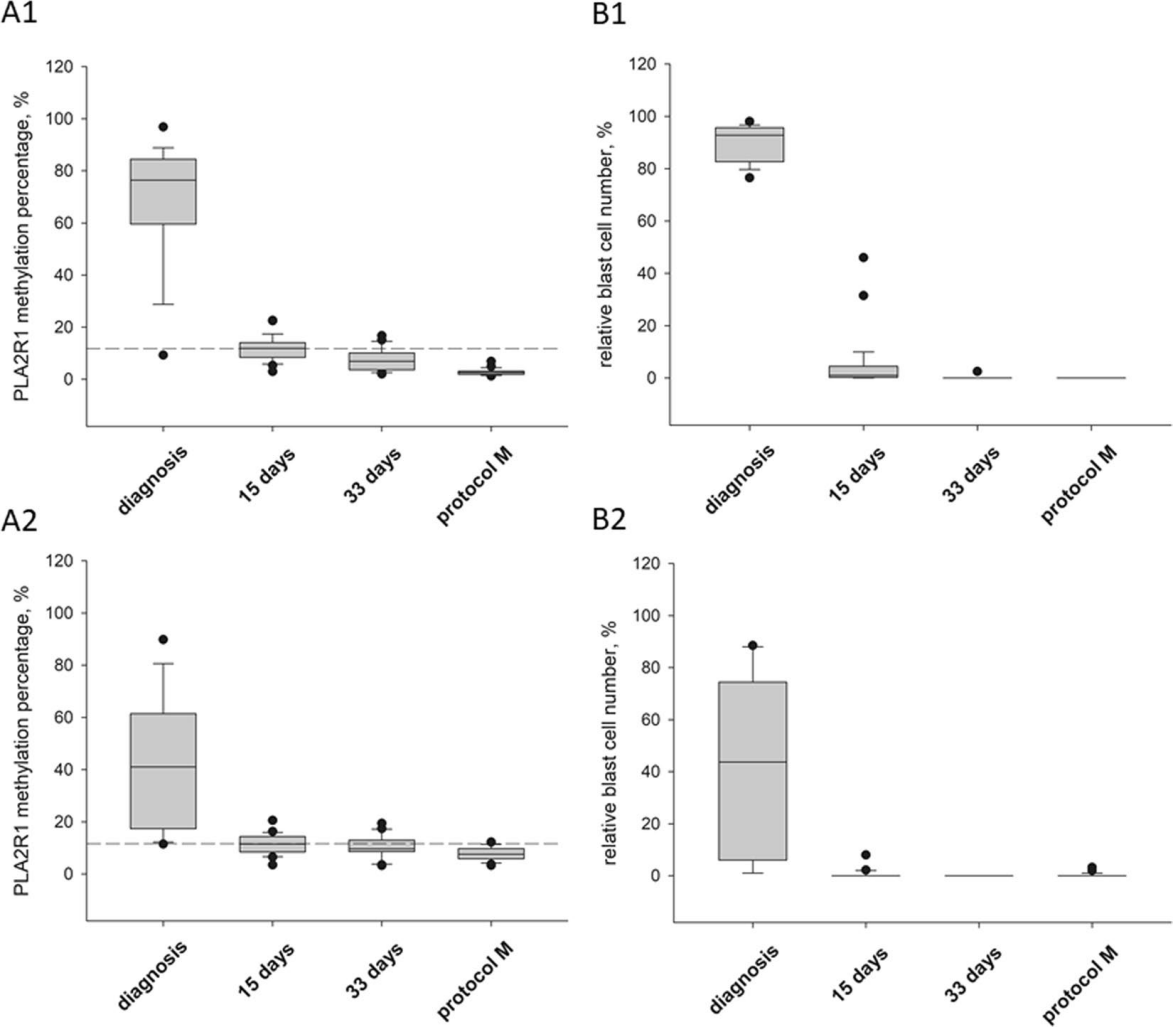
*PLA2R1* promoter methylation and blast cell occurrence during childhood ALL treatment protocol. Box plots consist of the median as ‘center value’, the 25^th^ and 75^th^ percentiles as box edges, and the 10th and 90th percentiles as whisker boundaries. (**A**) Percentage of *PLA2R1* promoter methylation was analysed using droplet digital PCR. (**B**) The relative blast cell number (number of blast cells in relation to the number of total leukocytes in %) was determined using light microscopic and flow cytometric analysis. Data were obtained for BM aspirates (A1, B1) and PB (A2, B2) from children with B cell ALL at diagnosis (n = 18), after 15 (n = 29) and 33 days (n = 22), and before protocol M (n = 29). Dashed lines indicate the 97.5^th^ percentile of the *PLA2R1* promoter methylation in the healthy control group.

**Figure 3:**
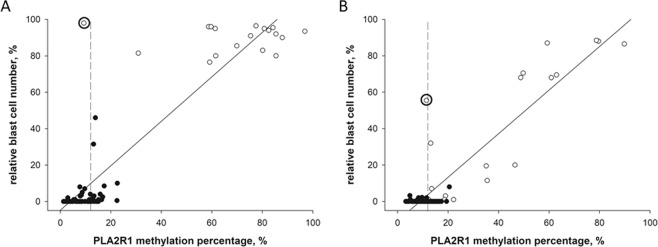
Linear correlations between *PLA2R1* promoter methylation and blast cell occurrence measured at the time of diagnosis and during childhood ALL treatment protocol. The scatter blots show the relative blast cell numbers plotted against the *PLA2R1* methylation percentages determined in BM aspirates (**A**) and PB samples (**B**) at the time of diagnosis (white dots) and following ALL treatment protocol (black dots). The percentage of *PLA2R1* promoter methylation was defined using ddPCR. The relative blast cell number (number of blast cells in relation to the number of total leukocytes in %) was determined using light microscopic and flow cytometric analyses. Significant positive correlations between the relative blast cell number and the *PLA2R1* methylation percentage were detected for BM aspirates (r = 0.92, p < 0.001, n = 98) and PB samples (r = 0.91, p < 0.001, n = 98). The patient with high relative blast cell numbers and low *PLA2R1* promoter methylation at diagnosis is indicated by bold circles for BM and PB samples. Dashed lines indicate the 97.5^th^ percentile of the *PLA2R1* promoter methylation in the healthy control group.

Corresponding PB samples exhibited, on average, approximately half the relative blast cell number of BM aspirates with 46.8% ± 32.3% (p = 0.032) and 48.5% ± 35.8% (p < 0.001) for pre-B and common ALL patients, respectively. In BM aspirates, all samples exhibited both strongly elevated relative blast cell numbers and increased levels of *PLA2R1* promoter methylation. A single exception was one patient with common ALL exhibiting 98% leukaemic blast cells but only 9.3% *PLA2R1* promoter methylation (Figs. 2 and 3).

### PLA2R1 promoter methylation and blast cell occurrence during ALL treatment protocol

During the treatment of patients, both the relative leukaemic blast cell number and the *PLA2R1* methylation degree decreased in parallel in PB and BM aspirates (Fig. 2A,B). *PLA2R1* promoter methylation and leukaemic blast cell number were analysed in BM aspirates and PB samples of children with B-cell ALL at defined timepoints of the ALL treatment protocol (Fig. 2). A representative example of *PLA2R1* promoter methylation quantitation by ddPCR and corresponding *PLA2R1* methylation percentages during the course of ALL treatment is shown in Supplementary Fig. 2. Two major FAM-positive subfractions were detected, indicating the presence of both homogenously and heterogeneously methylated epialleles identified by probe mismatching, resulting in different FAM signal amplitudes, as recently demonstrated^23^. Both the relative leukaemic blast cell number and the *PLA2R1* methylation percentages significantly decreased during the first 15 days of treatment compared to initial measurements at diagnosis. Mean *PLA2R1* promoter methylation of BM and PB samples decreased from 69.3% ± 20.9% to 11.8% ± 4.5% (p < 0.001, Fig. 2A1) and from 42.1% ± 25.0% to 11.4% ± 3.7% (p < 0.001), respectively (Fig. 2A2). The relative blast cell numbers decreased from 89.9% ± 6.7% to 5.0% ± 9.8% (p < 0.001, Fig. 2B1) and from 43.2% ± 34.3% to 0.4% ± 1.5% (p < 0.001, Fig. 2B2) in BM and PB samples, respectively. Both parameters declined continuously during ongoing ALL treatment. Minimal values were reached before the start of protocol M with 2.7% ± 1.2% or 7.7% ± 2.5% *PLA2R1* methylation and 0% or 0.2% ± 0.7% leukaemic blasts in BM aspirates or PB samples, respectively.

In conclusion, the correlation between *PLA2R1* promoter methylation and relative blast cell number was analysed in BM aspirates and PB samples at the time of diagnosis and during childhood ALL treatment protocol (Fig. 3). Both BM aspirates and PB samples exhibited significant positive correlations with correlation coefficients of 0.93 (p < 0.001) and 0.91 (p < 0.001), respectively.

### *PLA2R1* promoter methylation was increased in patients with ALL relapse

Five leukaemia relapses occurred during the present study period, comprising four B cell ALL cases and one patient with AML. Two representative examples of ALL relapse and the single case of AML relapse are presented in Supplementary Fig. 3. In BM aspirates with ALL and AML relapse, both *PLA2R1* promoter methylation percentages and leukaemic blast cell numbers were strongly elevated in the range of 68.3% ± 6.9% *PLA2R1* methylation (p < 0.001 compared to control group) and 87.2% ± 8.7% leukaemic blast cells. PB samples of ALL and AML relapse also exhibited an increase in *PLA2R1* methylation (27.1% ± 13.5%, p = 0.001) and relative blast cell numbers (21.3% ± 27.2%) in comparison to the control group. In BM and PB samples, *PLA2R1* methylation declined below the methylation level of the control group during the following ALL and AML treatment protocol (Supplementary Fig. 3).

### *PLA2R1* promoter methylation as biomarker for ALL risk stratification

**Figure 4:**
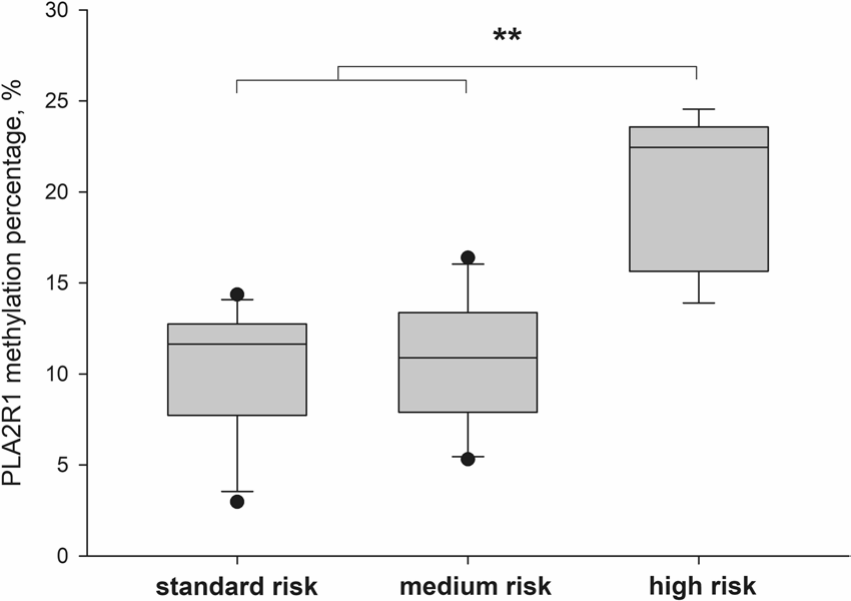
*PLA2R1* promoter methylation as biomarker for ALL risk stratification. Box plots consist of the median as ‘center value’, the 25^th^ and 75^th^ percentiles as box edges, and the 10th and 90th percentiles as whisker boundaries. The percentage of *PLA2R1* promoter methylation was analysed using droplet digital PCR. Data were obtained for BM aspirates from children with B cell ALL at day 15 of ALL induction treatment. Patients were subdivided into groups with standard (n = 11), medium (n = 15), and high risk (n = 5) according to the AIEOP-BFM ALL 2009 guidelines. The symbol **Indicates significant differences with p < 0.01 between marked cohorts.

The selection of the ALL treatment procedure and subsequent treatment success are dependent on the quality of risk stratification. In the present study, patients were subdivided into groups with standard, medium, and high risk according to the AIEOP-BFM ALL 2009 guidelines and the *PLA2R1* promoter methylation on day 15 of the ALL induction treatment was evaluated as biomarker for risk stratification (Fig. 4). The mean *PLA2R1* promoter methylation percentage of the standard and medium risk group were 10.0 ± 3.4 and 10.8 ± 3.5, respectively. *PLA2R1* promoter methylation of the high risk group was significantly increased with 20.2 ± 3.9 compared to the standard (p = 0.002) and medium risk group (p < 0.005; Fig. 4).

### Confirmation of the differential *PLA2R1* promoter methylation by Illumina Infinium 450k Human DNA methylation data analysis

The *PLA2R1* promoter section analysed in the present study is represented by three probes of the Illumina Infinium 450k Human DNA methylation Beadchip array (Supplementary Fig. 1). To validate our ddPCR results, three different GEO datasets (GSE49031^24^, GSE63409^25^, and GSE58477^26^) were analysed with regard to the methylation status of the distinct *PLA2R1* promoter region analysed in the present study. GSE49031 investigated the DNA methylation in paediatric ALL and included BM and PB samples of primary paediatric ALL taken at diagnosis (n = 764), remission (n = 86), and relapse (n = 32). Fractionated PB cells from healthy blood donors (n = 51) were also analysed. The methylation status of all *PLA2R*-associated Illumina probes is illustrated as heatmap in Supplementary Fig. 4 and as box plots in Supplementary Fig. 5. In BM ALL samples, *PLA2R1* promoter methylation was increased significantly at diagnosis (52% ± 22%) compared to corresponding samples at remission (10% ± 2%, p < 0.001). ALL relapse samples exhibited a significantly increased *PLA2R1* methylation of 65% ± 23% in comparison to samples taken from patients in remission (p < 0.001) and also compared to primary BCP-ALL (52% ± 22%), p < 0.001) and T-ALL (53% ± 24%), p = 0.02) samples (Supplementary Fig. 5A1). Moreover, *PLA2R1* methylation in PB ALL samples increased significantly compared to PB samples from healthy controls and their different cell fractions (p < 0.001, Supplementary Fig. 5A2).

Divergent methylation patterns of healthy progenitor cells were investigated in the GSE63409 and GSE58477 datasets and compared to leukaemic stem and blast cells of patients with AML (Supplementary Fig. 5B,C). Different types of normal BM-derived progenitor cells showed similar levels of *PLA2R1* promoter methylation of approximately 10%. By contrast, leukaemic progenitor cells exhibited a significantly higher degree of *PLA2R1* promoter methylation within the range of 30–50% (GSE63409: p < 0.001 for leukaemic stem cells or leukaemic blasts vs. healthy progenitor cells; GSE58477: p = 0.002 for normal CD34 + vs. leukaemic blasts).

Additionally, analyses of Illumina 450k methylation^27^ and gene expression data^28^ from six childhood B-ALL and eight childhood T-ALL cell lines demonstrated that the three Illumina 5’-CpG sites associated with the analysed ddPCR amplicon were strongly methylated. Simultaneously, the PLA2R1 expression was completely or at least notably suppressed in all analysed cell lines (Supplementary Table 2).

### Transfection-based overexpression of PLA2R1 in Jurkat cells

To further investigate the effect of different PLA2R1 expression levels in acute childhood leukaemia, human lymphocyte acute leukaemia (Jurkat) cells, which exhibit a high degree of *PLA2R1* promoter hypermethylation and an associated silenced receptor expression^19^, were analysed *in vitro*.

**Figure 5:**
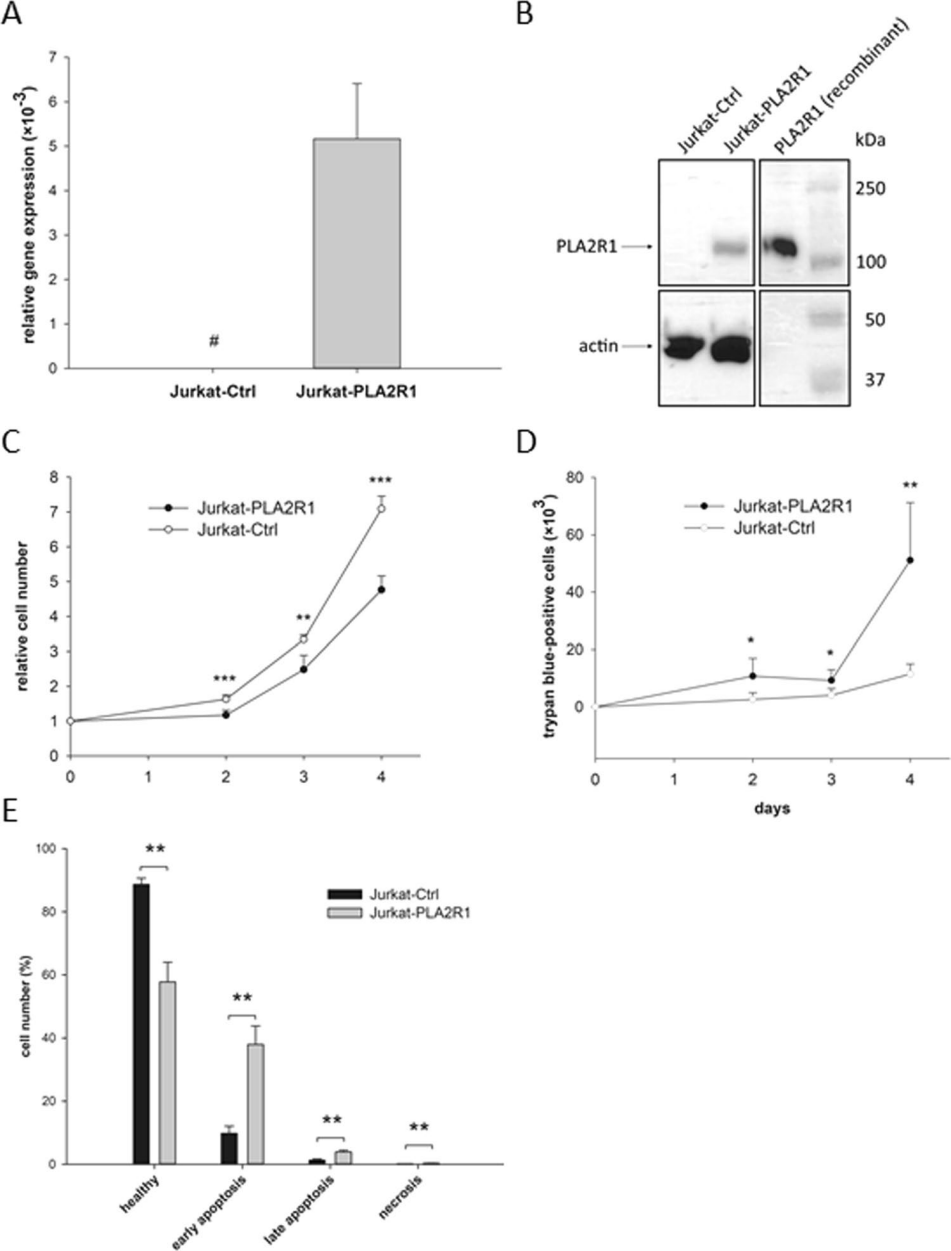
Cell proliferative behaviour in PLA2R1-transfected Jurkat cells (Jurkat-PLA2R1) compared to control vector-transfected Jurkat cells (Jurkat-Ctrl). (**A**) The amount of PLA2R1-mRNA was determined using RT-qPCR with β-actin as reference gene. Results are shown as mean ± SD of three independent experiments (biological n = 3) with two technical replicates. Bar graphs represent the ratio of PLA2R1 and β-actin gene expression. Symbol # indicates that PLA2R1 expression in Jurkat-Ctrl cells was not detected after 45 PCR cycles and therefore set to zero. (**B**) The level of PLA2R1 protein expression was assessed using western blotting with actin as reference protein and human recombinant PLA2R1 as positive control. A cropped representative blot section (from four independent experiments) is shown (biological n = 4, full-length blot is presented in Supplementary Fig. 6). (**C–E**) Results are the means ± SD of three independent experiments (biological n = 3) with two technical replicates. 2 × 10^5^ Jurkat cells were seeded in 6-well plates and trypan blue-negative (**C**) and -positive Jurkat cells (**D**) were counted during four consecutive days. (**E**) Apoptosis was stimulated with hydrogen peroxide for 24 h and determined by Annexin-V-Fluorescein/Hoechst 33258 staining using flow cytometry analysis. Levels of significance are defined as p < 0.05 (*), p < 0.01 (**), and p < 0.001 (***).

Jurkat cells were transfected with a *PLA2R1* plasmid vector (Jurkat-PLA2R1) to overexpress PLA2R1. Results were compared to control vector transfected Jurkat cells (Jurkat-Ctrl) (Fig. 5). Jurkat-Ctrl and Jurkat-PLA2R1 cells showed comparable levels of β-actin gene expression and actin protein synthesis indicated by quantitative real-time PCR and western blot analysis, respectively. A strong re-expression of PLA2R1 was demonstrated at mRNA and protein levels in Jurkat-PLA2R1 cells, whereas in Jurkat-Ctrl cells, no *PLA2R1* gene expression and protein synthesis were detectable (Fig. 5A,B; uncropped western blotting displayed in Supplementary Fig. 6).

### PLA2R1 expression influences the proliferative behaviour of Jurkat cells

The proliferation rate of transfected Jurkat cells was assessed using cell growth curve analysis with trypan blue staining of dead cells (Fig. 5C,D). The relative cell number of Jurkat-PLA2R1 was significantly decreased in comparison to Jurkat-Ctrl cells after two days of culturing (p < 0.001) and observed differences increased continuously over the investigation period (Fig. 5C). The maximum change in Jurkat-PLA2R1 cell proliferation was reached after four days, with 33% reduction compared to Jurkat-Ctrl cells (p < 0.001). Furthermore, the number of dead cells was significantly increased in Jurkat-PLA2R1 cells after two days of culture compared to Jurkat-Ctrl cells (p = 0.015), reaching a 4.5-fold higher difference after four days (p = 0.002, Fig. 5D).

Moreover, the transfected Jurkat cells were stained with Annexin-V-Fluorescein/Hoechst 33258 and analysed by flow cytometry to investigate the underlying mechanism of cell death (Fig. 5E). Representative flow cytometry results of transfected Jurkat cells are presented in Supplementary Fig. 7. Jurkat-PLA2R1 exhibited 31% fewer healthy cells than Jurkat-Ctrl (p = 0.003), whereas the fractions of early (p = 0.003) and late apoptotic (p = 0.004) and necrotic cells (p = 0.003) were significantly increased in Jurkat-PLA2R1 compared to Jurkat-Ctrl cells after induction of cell death with hydrogen peroxide.

## Discussion

The present study establishes that in addition to adult patients with AML^19^, the *PLA2R1* promoter is also hypermethylated, both in BM aspirates and PB samples from paediatric patients with primary and relapsed acute lymphoblastic leukaemia. Furthermore, lower *PLA2R1* promoter methylation coincided with decreases in blast cell numbers in BM and PB during ALL induction treatment. Significant positive correlations between *PLA2R1* promoter methylation and relative blast cell numbers were detected in BM and PB samples during complete ALL treatment protocol. Therefore, our data suggests that leukaemic blasts are the origin of *PLA2R1* hypermethylation in BM and PB samples.

Although the number of blasts was above 76% in all analysed BM aspirates at diagnosis, *PLA2R1* promoter methylation levels varied between 31% and 97%. This data suggests that not all BM cells defined as blasts had methylated *PLA2R1* promoter sequences. The observed data indicates BM blast cell heterogeneity, which is consistent with previous data^29–31^. Moreover, normal BM precursors of mature B-lymphocytes may be present within the analysed aspirates. The precursors share many morphological and immunophenotypic features with neoplastic lymphoblasts of B-ALL^32^. According to our analysis of Illumina Infinium 450k Human DNA methylation data, normal BM precursors exhibit only marginal *PLA2R1* promoter methylation (Supplementary Fig. 5). Therefore, a different proportion of precursors may predispose BM blasts to greater variation in *PLA2R1* methylation compared to PB. Further studies involving cell sorting experiments are required to verify this hypothesis.

Taken together, our findings support the hypothesis, initially established from the obtained data in adult patients with AML^19^, that the determination of *PLA2R1* methylation may be also a sensitive marker for treatment efficacy and monitoring of minimal residual disease in childhood ALL. *PLA2R1* methylation can be of special interest in patients lacking a common leukaemia subtype classification if no other molecular biomarkers are available. One example of ALL relapse in a patient without ALL-specific molecular genetic markers is shown in Supplementary Fig. 3A (left diagram). In this case unknown ALL-specific genetic markers limited the monitoring of minimal residual disease and therapy effectivity. Generally, *PLA2R1* promoter methylation was found to be strongly elevated at diagnosis in BM and PB samples but decreased rapidly during ALL induction treatment mostly falling below the methylation degree of healthy controls. Furthermore, *PLA2R1* promoter methylation was significantly increased in the group of high risk patients compared to non-high risk groups at day 15 of the ALL induction treatment. Risk stratification according to the AIEOP-BFM ALL 2009 guidelines is a multi-parameter approach, involving immunohistochemical as well as molecular biological analyses at different points in time (until week 12) of the ALL treatment protocol. Thus, *PLA2R1* promoter methylation may be beneficial to identify high risk patients relatively easily, enabling early adaptions to the ALL treatment protocol. To what extend monitoring of minimal residual disease by *PLA2R1* promoter methylation is superior to well established methods using clonal markers like IgG-heavy chain or T-cell receptor rearrangements will be the next step of our analysis. In this context it must be kept in mind that unlike chromosomal alterations, gene mutations and gene rearrangements, promoter hypermethylation is reversible and may be altered by therapeutic intervention. There are existing approaches, including the introduction of epigenetic modifying agents into leukaemia treatment protocols^33^, and *PLA2R1* promoter methylation may be an indicator to predict the effectiveness of such treatment strategies^19^.

In this cohort of paediatric patients, one patient with common ALL exhibited strongly increased relative leukaemic blast cell numbers together with a lack of increased *PLA2R1* promoter methylation in both BM and PB samples at diagnosis, suggesting that *PLA2R1* promoter hypermethylation is a frequent (95%, 19 out of 20 B-cell ALL patients), but not indispensable event during childhood leukaemogenesis. These findings are consistent with the work of Amin *et al*., suggesting the existence of a subset of AML and ALL patients with PLA2R1-expressing leukaemic blast cells^34^. This may correspond to the aforementioned ALL patient exhibiting *PLA2R1* promoter methylation degree below the cutoff of the healthy cohort.

The obtained *in vitro* data from *PLA2R1*-transfected Jurkat cells suggest that blast cells with silenced PLA2R1 expression may escape apoptosis and necrosis more efficiently than the receptor expressing cells. These findings indicate that PLA2R1 has a tumour-suppressive effect in acute leukaemia. We previously provided evidence of PLA2R1’s tumour-suppressive role in solid tumours investigating the prostate cancer cell line LNCaP in *in vivo* xenograft models^35^. Re-expression of the receptor was also discussed and shown to have a tumour-suppressive function in the mammary cancer cell line MDA-MB-453, mediated by JAK2/oestrogen-related receptor-α signalling and the induction of mitochondrial apoptosis pathways^15,16,35^. It may be possible to activate similar pro-apoptotic signalling pathways in *PLA2R1*-transfected Jurkat cells. However, this suggestion requires further investigation.

To further evaluate the relevance of our results, we searched the methylation data published by Nordlund *et al*. using the Illumina Infinium 450k Human DNA methylation Beadchip array^24^. Our analysis confirmed an increased methylation level of the *PLA2R1* promoter in BM and PB samples of patients with childhood ALL compared to matched samples of patients in remission or healthy blood donors. Furthermore, our analysis suggested an increase of *PLA2R1* promoter methylation in BM aspirates of patients with ALL relapse compared to primary samples. This is in line with the strongly elevated *PLA2R1* promoter methylation in cases of childhood ALL relapse as investigated in the present study.

Moreover, the analysis of Illumina DNA methylation results from patients with AML demonstrated that isolated leukaemic progenitor cells exhibited high degrees of *PLA2R1* promoter methylation, whereas normal progenitor cells showed only minor *PLA2R1* methylation (Supplementary Fig. 5). These results are consistent with our conclusion that leukaemic blast cells are the origin of the increased *PLA2R1* methylation observed in this study of paediatric patients with ALL.

A limitation of our study is the selection of the Jurkat T-ALL cell line as the cell model for our transfection experiments, as it may limit the significance of the *in vitro* results to children diagnosed with B-ALL. Therefore, further investigation is warranted into whether the tumour-suppressive behaviours of PLA2R1 re-expression will be repeated in B-ALL cell lines and primary blast cells. Using isolated blast cells, it will be possible to determine the extent to which the *PLA2R1* gene is actually silenced in blasts of childhood ALL patients at the mRNA and protein levels. In the current study, we have shown that the three Illumina 5′-CpG sites associated with the analysed ddPCR amplicon were strongly methylated and that PLA2R1 expression was completely or notably suppressed in six childhood B-ALL and eight T-ALL cell lines, including the investigated Jurkat cell line (Supplementary Table 2). The reproducibility of these analyses was also supported by the data observed in U937, PC-3, and LNCaP cells. These results were consistent with our previous sequencing data for Jurkat and U937 cells, as well as for the prostate cancer cell lines^18,19,35^.

## Conclusion

The *PLA2R1* promoter was found to be hypermethylated in BM aspirates and PB samples of patients diagnosed with different childhood ALL subtypes, which makes the *PLA2R1* methylation a suitable biomarker for the monitoring of treatment response in ALL patients. A decline in the leukaemic blast cell numbers together with decreasing *PLA2R1* promoter methylation during ongoing ALL treatment suggest that the overall increase of *PLA2R1* methylation in BM and PB samples at diagnosis is caused by leukaemic blast cells. Furthermore, the data of this study demonstrated that *PLA2R1* promoter methylation may be a useful marker for disease monitoring, risk stratification, and the evaluation of ALL treatment success. This is especially relevant to patients lacking known ALL-specific molecular genetic markers for minimal residual disease monitoring or to predict the effectiveness of epigenetic modifying agents in ALL treatment. *In vitro* analysis showed that the *PLA2R1* re-expression and up-regulation of protein synthesis in transfected Jurkat cells had a negative impact on cellular proliferative behaviour, suggesting an important tumour-suppressive role of PLA2R1 in childhood ALL.

## Material and Methods

### Sample collection

This study included 44 patients with B-lymphoblastic ALL. Additionally, a single patient with acute myeloid leukaemia (AML; 13 years old male) was assessed for comparison. The B-cell ALL cohort consisted of five cases with pre-B ALL (four females and one male) and 39 cases with common ALL (15 females, 24 males). The average age was 3.0 ± 1.1 years (range 1.1 to 4.0 years) and 7.5 ± 4.4 (range 1.7 to 17.0 years), respectively. EDTA-bone marrow and peripheral EDTA-blood samples were collected at routine examinations. EDTA-peripheral blood was collected from 20 healthy individuals (8 females and 12 males) with average ages of 9.4 ± 4.2 years (range 3.3 to 16.2 years), serving as controls. For risk stratification, bone marrow aspirates from day 15 of the ALL induction treatment were subdivided into groups with standard (n = 11), medium (n = 15), and high risk (n = 5) according to the AIEOP-BFM ALL 2009 guidelines based on chromosomal rearrangements present at diagnosis, prednisolone treatment response at day 8 and overall treatment response assessed in bone marrow aspirates at day 15 of the ALL treatment protocol. An overview of patients and healthy control samples included in the present study is presented in the Supplementary Table 1. The relative blast cell numbers (number of blast cells in relation to the number of total leukocytes in %) were routinely quantified at diagnosis and during ALL and AML treatment protocol by light microscopy and flow cytometry analysis. All patients and their legal guardians provided written informed consent for the study. The study was approved by the Ethical Board of the University Hospital of Dresden. All methods were performed in accordance with the relevant guidelines and regulations.

### Extraction of genomic DNA and bisulfite modification

Genomic DNA was isolated from peripheral blood (PB) and bone marrow (BM) aspirate cells as described^36^ and precipitated in ethanol. Concentration of isolated DNA was measured using the Quantifluor dsDNA-System (Promega GmbH, Mannheim, Germany) according to the manufacturer’s protocol. Bisulfite modification was conducted with 500 ng of isolated genomic DNA using the EZ DNA Methylation Kit (Zymo Research Europe GmbH, Freiburg, Germany).

### Digital droplet PCR of bisulfite-modified DNA

The methylation status of the *PLA2R1* promoter and down-stream regions −700 to +1340 bp relative to the transcription start site (TSS) was previously analysed in different leukaemic cell lines and in PB samples as well as BM aspirates of adult leukaemic patients via methylation-sensitive high-resolution melting^19^. Three different 5′-CpG sites (−546 bp, −548 bp and −551 bp relative to TSS) were selected by maximum methylation differences between healthy individuals and leukaemic patient samples/cell lines to represent the methylation status of the distinct region −644 bp to −477 bp from the TSS of *PLA2R1*^19,23^ (Supplementary Fig. 1). Digital droplet PCR protocol was performed as described elsewhere^23^ and according to the manufacturer’s instructions (Bio-Rad GmbH, München, Germany). Binding of dual-labelled probes was used to simultaneously quantify methylated (5′-FAM-CCCAACTACTCCGCGACGCAA-3′-BHQ1) and unmethylated (5′-HEX-CCCAACTACTCCACAACACAA-3′-BHQ1) DNA fragments. The applied primer pair 5′-GGG GTA AGG AAG GTG GAG AT-3′ and 5′-ACA AAC CAC CTA AAT TCT AAT AAA CAC-3′ (168 bp product length) was designed to anneal to methylation-independent sections of the DNA. Data were processed using the QuantaSoft software version 1.6.6.0320 (Bio-Rad GmbH). The *PLA2R1* methylation percentage was calculated using the number of methylated copies in relation to the sum of methylated and unmethylated *PLA2R1* copies in %.

### Cell culture and incubation

Human T-lymphocyte acute leukaemia (Jurkat) cells were purchased from the German Collection of Microorganisms and Cell Cultures (DSMZ, Braunschweig, Germany) and cultured as previously described^19,35^. Transfected Jurkat cells were cultured in RPMI-1640 medium (Thermo Fisher Scientific, Waltham, MA, USA) supplemented with 10% heat-inactivated fetal calf serum (Thermo Fisher Scientific) and 500 mg/ml G418 Sulfate (Thermo Fisher Scientific). Cell lines were frequently tested for mycoplasma contamination (MycoAlert™ Mycoplasma Detection Kit, Lonza).

### Transfection

According to our previous studies with lymphocytic Jurkat cell line^19^, a fully methylated *PLA2R1* promoter was detected using sequencing analysis in conjunction with PLA2R1 gene silencing. The methylation resulted in a lack of PLA2R1 expression at both mRNA and protein level. Furthermore, a strong re-expression of PLA2R1 was observed after azacitidine (Vidaza) treatment of Jurkat cells *in vitro*. Therefore, we selected Jurkat cell line as a relevant cell model for our transfection experiments. This established cell line is extensively used as cell model to investigate ALL^20–22^.

Transfection of Jurkat cells was performed as described elsewhere^35^ utilizing ViaFect^TM^ Transfection Reagent (Promega, Madison, WI, USA) and an optimized expression vector for phospholipase A2 receptor 1 (NP_031392.3; GenScript, Piscataway, NJ, USA), or a control vector (GenScript). The transfected cells were selected using 500 mg/mL G418 Sulfate and surviving cells were used for subsequent experiments.

### RNA isolation and reverse transcription quantitative polymerase chain reaction (RT-qPCR)

RNA isolation and RT-qPCR were described elsewhere^35^. In brief, RNA was isolated using QIAzol Lysis Reagent (QIAGEN) and miRNeasy Mini Kit with on-column DNase digestion (QIAGEN). Reverse transcription protocol involved incubation of 100 ng RNA template and MuLV Reverse Transcriptase (Thermo Fisher Scientific) for 45 min at 42 °C and 5 min at 99 °C with a final reaction volume of 20 µL. Quantitative PCR was conducted using the Rotor-Gene Q cycler and Rotor-Gene SYBR Green PCR Kit (Qiagen). PCR conditions involved 5 min at 95 °C followed by 45 cycles of 5 s at 95 °C, and 10 s at 59 °C. β-actin was used as reference gene. RNA isolation and RT-qPCR were carried out using three separate cell culture passages, each measured in duplicate (biological n = 3, technical n = 2).

### Protein isolation, SDS-PAGE and Western Blot

Protein isolation from cell culture experiments, SDS-PAGE, and Western Blot were described elsewhere^35^. The method involves cell lysis with CellLytic M lysis buffer (Sigma-Aldrich), protein separation using ClearPAGE 10% sodium dodecylsulfate polyacrylamide gels (C.B.S. Scientific), and sample transfer to PVDF membranes (Fisher Scientific). For detection of PLA2R1, primary recombinant rabbit monoclonal antibody (AB; ab211573, Abcam, Cambridge, UK) and a secondary horseradish peroxidase conjugated goat anti-rabbit IgG (sc-2004, Santa Cruz) were used. Actin was detected using primary mouse monoclonal AB (MAB1501R, Merck Millipore, Darmstadt, Germany) and secondary horseradish peroxidase conjugated goat anti-mouse IgG (sc-2005, Santa Cruz). Protein isolation, SDS-PAGE and Western Blot were repeated three times using separate cell culture passages (biological n = 4).

### Analysis of proliferation and apoptosis/necrosis

For cell growth curve analysis, 2 × 10^5^ transfected Jurkat cells were cultured over four days. Numbers of living and dead cells were determined on the second, third and fourth day by trypan blue staining and light microscopic analysis.

Cell death was stimulated with 100 µM hydrogen peroxide (Sigma-Aldrich) in RPMI-1640 medium without fetal calf serum for 24 h. The cells were stained with the Annexin-V-FLUOS staining kit (Sigma-Aldrich) according to the manufacturer’s protocol. Necrotic cells were stained with Hoechst 33258 (Thermo Fisher Scientific) for 30 s and analysed immediately afterwards using flow cytometry to avoid the staining of healthy cells.

### Data analysis

‘Center values’ were defined as means with standard deviation as error indication. Normal distribution was analysed using the Shapiro-Wilk test. Variance was estimated using F-test. Data were analysed by two-tailed and unpaired Student’s *t* test (normally distributed, homoscedastic) or Mann-Whitney Rank Sum test (non-normally distributed) to calculate the indicated *p* values. Differences were considered significant at *p* < 0.05. Levels of significance were defined and indicated as *p* < 0.05 (*), *p* < 0.01 (**), and *p* < 0.001 (***).

The Illumina Infinium 450 k Human DNA methylation Beadchip array data was calculated as normalized β-values from four different Gene Expression Omnibus (GEO) datasets. The specific normalization methods were described previously (GSE49031^24^, GSE58477^25^, GSE63409^26^, and GSE68379^27^). For the GSE49031 dataset, methylation raw data were normalized using peak-based correction, whereas GSE58477, GSE63409, and GSE68379 datasets were normalized using appropriate processing software (Illumina^®^ GenomeStudio^®^ for the first and minfi Bioconductor package for the remaining two datasets). RefSeq gene annotations for Illumina 5′-CpG sites were based on the Human Methylation 450 k manifest file version 1.2 (May 23, 2013). Illumina probes with *PLA2R1* annotation were filtered. The remaining probes associated with *PLA2R1* were again filtered according to the latest Illumina annotation file^37^, excluding probes with INDELs, annotated single-nucleotide polymorphism (SNP) at the single-base extension or CpG sites, and non-unique subsequences within the probe sequence. Four out of 18 Illumina 5′-CpG sites were excluded based on the aforementioned criteria. According to the annotated UCSC gene region feature categories, the Illumina Infinium 450 k Human DNA methylation Beadchip array includes five CpG sites within the body of *PLA2R1* as well as five CpG sites within 0–200 bases (TSS200), and four CpG sites within 200–1500 bases (TSS1500) upstream of the transcriptional start site. The three different CpG sites associated with the analysed 168 bp ddPCR amplicon are associated with the latter category. Average methylation was estimated as mean β-values for the three different 5′-CpG sites (cg12991125, cg20257553, and cg24235037), that are located within the analysed ddPCR amplicon (Supplementary Fig. 1).

For analysis of gene expression, processed data of leukaemic cell lines were provided by the Cancer Cell Line Encyclopedia (CCLE) project^28^. In brief, for large insert non-strand specific RNA Sequencing, samples were processed according to Illumina Tru Seq RNA Sample Preparation protocol with 200 ng of total RNA as starting material. Illumina sequencing was done on the Illumina HiSeq. 2000 or HiSeq. 2500. RNA sequencing results are shown as TPM (Transcripts Per Kilobase Million).

### Ethics approval and consent to participate

All patients and their legal guardians provided written informed consent for the study. The study was approved by the Ethical Board of the Universxity Hospital of Dresden.Name of the registry: Eudra-CT; trial registration number: 2007-004270-43; date of the registration: 03/12/2010; https://www.clinicaltrialsregister.eu/ctr-search/trial/2007-004270-43/AT.

### Consent for publication

All patients and their legal guardians provided written informed consent for the publication of this study.

## Supplementary information

Supplementary Information.

## Data Availability

The datasets generated and analysed during the current study are not publicly available due to protection of privacy of the participants of the study but are available from the corresponding author on reasonable request.
