# Supplementary material for: Methylation of the Phospholipase A2 Receptor 1 Promoter Region in Childhood B Cell Acute Lymphoblastic Leukaemia
Source: Sci Rep. 2020 Jun 3;10:9058. doi: 10.1038/s41598-020-65825-0 (PMC7270080; doi:10.1038/s41598-020-65825-0)
Supplement: Supplementary file 1 — Supplementary Information. [file 41598_2020_65825_MOESM1_ESM.pdf]

Methylation of the Phospholipase A2 Receptor 1 Promoter Region in Childhood B Cell Acute Lymphoblastic Leukaemia

Markus Friedemann[1], Katharina Gutewort[1], Dana Thiem[1], Brit Nacke[1], Carsten Jandeck[1], Björn Sönke Lange[2], Olga Sukocheva[3], Meinolf Suttorp[4], Mario Menschikowski[1*]

[1]*Institute of Clinical Chemistry and Laboratory Medicine, University Hospital Carl Gustav Carus, Technical University of Dresden, 01307 Dresden, Germany*

[2]*Department of Paediatrics, University Hospital Carl Gustav Carus, Technical University of Dresden, 01307 Dresden, Germany*

[3]*School of Health Sciences, Flinders University of South Australia, Bedford Park, 5042, Australia*

[4]*Medical Faculty, Paediatric Haemato-Oncology, Technical University, 01307 Dresden, Germany*

*Correspondences should be addressed to:

Prof. Dr. Mario Menschikowski, Fetscherstraße 74, D-01307 Dresden, Germany, Phone: +49-351-458-2634; Fax: +49-351-458-4332; e-mail: Mario.Menschikowski@uniklinikum-dresden.de

Supplementary information

Supplementary Table 1. Characteristics of acute childhood leukaemia patients and healthy controls included in the current study. Patients were subdivided into standard (SR), medium (MR), and high risk (HR) groups according to the AIEOP-BFM ALL 2009 guidelines based on chromosomal rearrangements present at diagnosis, prednisolone treatment response at day 8 and overall treatment response assessed in bone marrow aspirates at day 15 of the ALL treatment protocol.

Supplementary Table 2. PLA2R1 gene expression and promoter methylation levels are summarized for different childhood acute leukaemia cell lines. PLA2R1 gene expression was analysed by Illumina RNA sequencing [28]. RNA sequencing results are presented as TPM (Transcripts Per Kilobase Million). *PLA2R1* promoter methylation levels were analysed using Illumina Infinium 450k Human DNA methylation array [27] The methylation beta values are shown for the three Illumina CpG sites covered by the analysed ddPCR amplicon (cg12991125, cg20257553, and cg24235037), as illustrated in Suppl. Fig. 1. The adult acute monocytic leukaemia cell line U937 and the prostate carcinoma cell lines LNCaP and PC-3 are listed below as a comparison. U937 and LNCaP cells exhibited *PLA2R1* promoter hypermethylation and silenced *PLA2R1* expression, whereas PC-3 exhibited a minor degree of *PLA2R1* promoter methylation and endogenous PLA2R1 expression [19,35]. The colon adenocarcinoma cell line SW948 was listed as an example of low degree of *PLA2R1* promoter methylation and high levels of *PLA2R1* gene expression.

Supplementary Figure 1. Schematic overview of the *PLA2R1* promoter region. Numbers indicate genomic position relative to the *PLA2R1* transcription start site (TSS). Rectangles represent methylation-independent primers (white) and the ddPCR probe (grey). Circles symbolize 5'-CpG sites within the 168 bp PCR amplicon. Methylation status of the 5'-CpG

sites 1-3 was detected by ddPCR probes. Dashed circles indicate 5'-CpG sites included in the Illumina Infinium 450k Human DNA methylation array.

Supplementary Figure 2. *PLA2R1* promoter methylation analysis using ddPCR during childhood ALL treatment. (A) One representative example of normalized ddPCR results from BM aspirates (A1) and PB samples (A2) of a common ALL patient at diagnosis, after 15 and 33 days and before protocol M is shown. (B) Corresponding *PLA2R1* promoter methylation percentages of BM and PB samples during ALL treatment protocol.

Supplementary Figure 3. *PLA2R1* promoter methylation of individual cases of ALL and AML relapse. Percentage of *PLA2R1* promoter methylation was analysed in two cases of ALL relapse (A) and one individual case with diagnosed AML relapse (B) using droplet digital PCR. Dashed lines indicate the 97.5th percentile of the *PLA2R1* promoter methylation in the healthy control group. Dotted lines represent the time of leukaemia relapse diagnosis. The arrow in (B) indicates a second, black dot in addition to the white dot.

Supplementary Figure 4. Heatmaps of *PLA2R1* methylation analyses in normal and leukaemic cells. Normalized data were obtained from studies conducted by Nordlund *et al.* (GSE49031) with BM (A) and PB samples (B) of childhood ALL [24]. The heatmap is based on the β-values of different samples. Higher β-values represent higher methylation (red), whereas lower β-values reflect lower methylation (green). (A) BM samples correspond to B-cell precursor (BCP-ALL) or T-cell ALL samples (T-ALL) at ALL diagnosis, remission, and relapse of leukaemia. (B) PB cells from healthy blood donors were analysed together as mixture (WB) or were sorted using cell surface markers $CD19^+$ (B cells), $CD3^+$ (T cells), or $CD34^+$ (stem cells). The Illumina Infinium 450k Human DNA methylation Beadchip array includes five CpG sites within the body of *PLA2R1* as well as five CpG sites within 0 - 200 bases (TSS200), and four CpG sites within 200 - 1500 bases (TSS1500) upstream of the transcriptional start site. The corresponding UCSC gene region feature categories are indicated

above. The three different CpG sites associated with the analysed 168 bp ddPCR amplicon are labelled in red.

Supplementary Figure 5. *PLA2R1* promoter methylation analysis of different normal and leukaemic cell types. Box plots consist of the median as ‘center value’, the 25th and 75th percentiles as box edges, and the 10th and 90th percentiles as whisker boundaries. The Illumina Infinium 450k Human DNA methylation Beadchip array includes three different 5’-CpG sites within the analysed 168 bp ddPCR amplicon (Supplementary Fig. 1). (A) Data were analysed using studies of Nordlund *et al.* (GSE49031) for BM (A1) and PB samples (A2) of childhood ALL [24]. (A1) BM samples were taken from B-cell precursor (BCP-ALL) or T-cell ALL samples (T-ALL) at ALL diagnosis, remission, and cancer relapse. (A2) PB cells of healthy blood donors were analysed together as mixture (WB) or were sorted by cell surface markers CD19$^+$ (B cells), CD3$^+$ (T cells), or CD34$^+$ (stem cells). *PLA2R1* promoter methylation was compared with BCP-ALL and T-ALL samples at diagnosis. (B) *PLA2R1* promoter methylation of normal hematopoietic stem and progenitor cells – common myeloid (CMP), granulocyte-macrophage (GMP), late-multipotent (L-MPP), megakaryocyte-erythroid (MEP), multipotent progenitor cells (MPP), and hematopoietic stem cells (HSC) - was compared with the methylation status of leukaemic stem (LSC) and blast cells of patients BM with AML (GSE63409) [25]. (C) *PLA2R1* methylation of leukaemia stem/blast cells was obtained from AML patients BM and compared to normal hematopoietic stem/progenitor cells from normal BM (GSE58477) [26]. Symbol * indicates significant differences with $p < 0.05$.

Supplementary Figure 6. Western blot raw data of transfected Jurkat cells.

Supplementary Figure 7. Apoptosis/necrosis in PLA2R1-transfected Jurkat cells (Jurkat-PLA2R1) compared to control vector-transfected Jurkat cells (Jurkat-Ctrl). Cell death was stimulated by hydrogen peroxide for 24 h and determined by Annexin-V-

Fluorescein/Hoechst 33258 staining and flow cytometry analysis in Jurkat-Ctrl (A) and Jurkat-PLA2R1 cells (B). Representative graphs are shown (n=3).

Supplementary Table 1

	Control	Pre-B ALL	Common ALL	AML
n	20	5	39	1
Average age [years]	9.4 ± 4.2	3.0 ± 1.1	7.5 ± 4.4	13
Minimum age	3.3	1.1	1.7	-
Maximum age	16.2	4.0	17.0	-
AIEOP-BFM ALL 2009 risk stratification	-	- 3 MR<br>- 2 HR	- 11 SR<br>- 20 MR<br>- 4 HR<br>- 4 relapse	-
Genotype	-	- 4 patients with no typical rearrangements (TEL/AML1, MLL, BCR/ABL)<br>- 1 patients TEL/AML1 positive	- 28 patients with no typical rearrangements (TEL/AML1, MLL, BCR/ABL)<br>- 10 patients TEL/AML1 positive<br>- 1 patient with t(2;14) translocation and IGH locus involvement	-

Supplementary Table 2

Cell line	Disease	PLA2R1 gene expression (TPM)	*PLA2R1* methylation (β-value)		
			cg12991125	cg20257553	cg24235037
697	childhood B acute lymphoblastic leukaemia	no expression	0.973	0.960	0.874
KOPN-8	childhood B acute lymphoblastic leukaemia	no expression	0.454	0.966	0.888
MHH-CALL-2	childhood B acute lymphoblastic leukaemia	no expression	0.525	0.954	0.230
MHH-CALL-4	childhood B acute lymphoblastic leukaemia	no expression	0.942	0.924	0.730
RCH-ACV	childhood B acute lymphoblastic leukaemia	no expression	0.869	0.926	0.685
SUP-B15	childhood B acute lymphoblastic leukaemia	0.1	0.983	0.977	0.906
ALL-SIL	childhood T acute lymphoblastic leukaemia	no expression	0.971	0.936	0.884
DND-41	childhood T acute lymphoblastic leukaemia	0.1	0.980	0.936	0.875
JURKAT	childhood T acute lymphoblastic leukaemia	no expression	0.985	0.962	0.806
MOLT-13	childhood T acute	0.3	0.973	0.973	0.886

	lymphoblastic leukaemia				
MOLT-16	childhood T acute lymphoblastic leukaemia	no expression	0.969	0.931	0.868
P12-ICHIKAWA	childhood T acute lymphoblastic leukaemia	0.1	0.980	0.976	0.878
PF-382	childhood T acute lymphoblastic leukaemia	0.1	0.985	0.938	0.879
RPMI-8402	childhood T acute lymphoblastic leukaemia	no expression	0.985	0.974	0.879
U-937	adult acute monocytic leukaemia	no expression	0.925	0.896	0.770
LNCaP	prostate carcinoma	no expression	0.748	0.830	0.463
PC-3	prostate carcinoma	3	0.074	0.224	0.186
SW948	colon adenocarcinoma	41	0.067	0.179	0.047

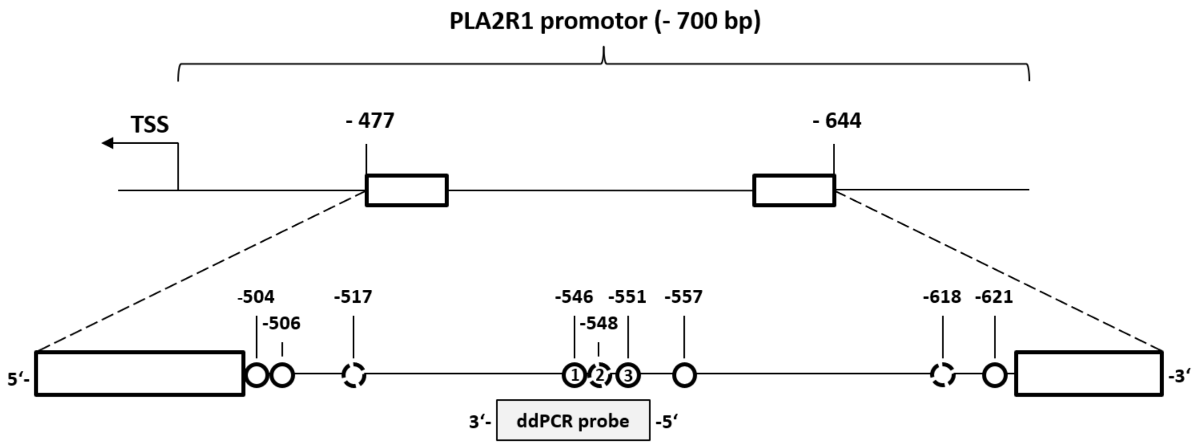

Supplementary Figure 1

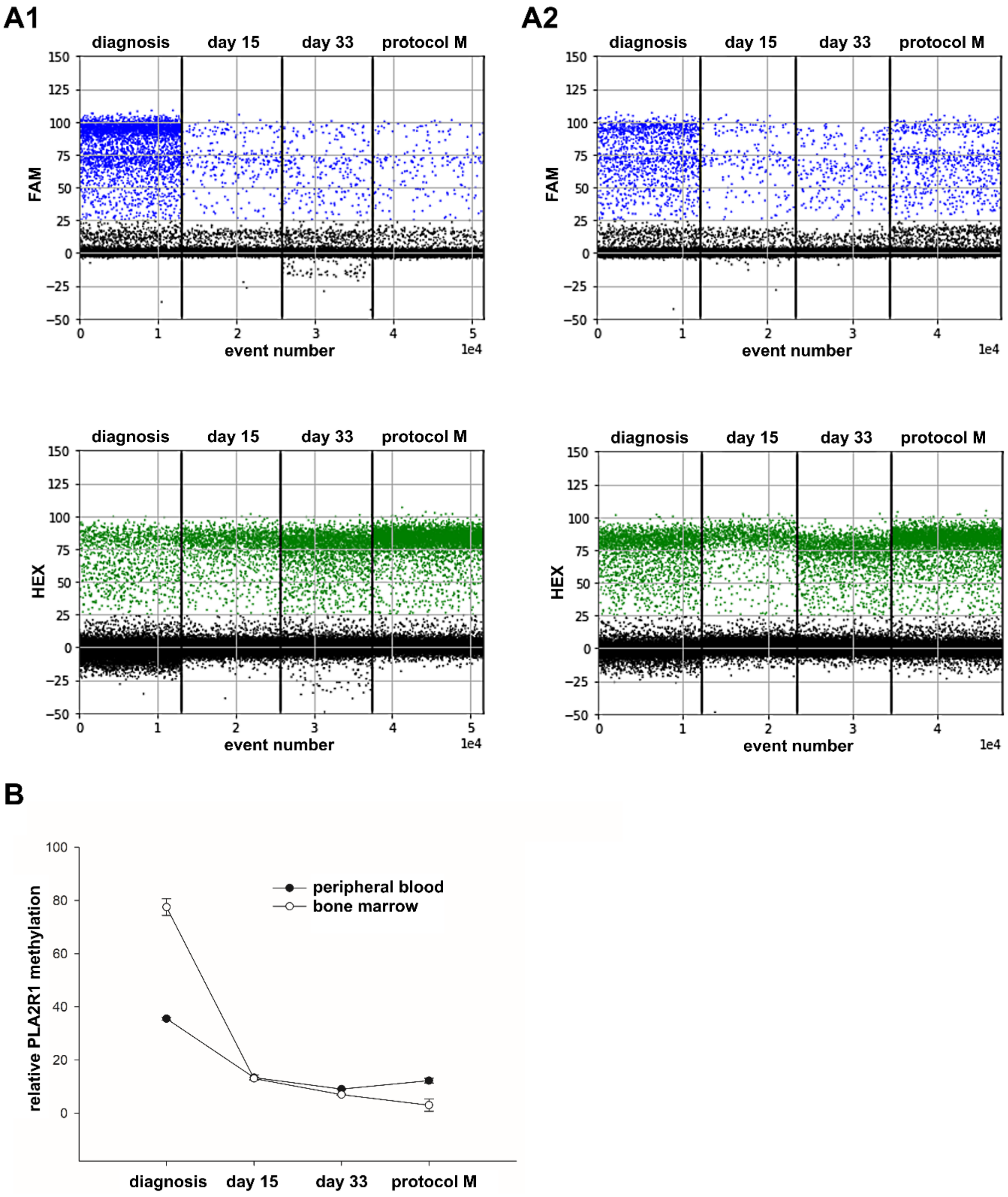

Supplementary Figure 2

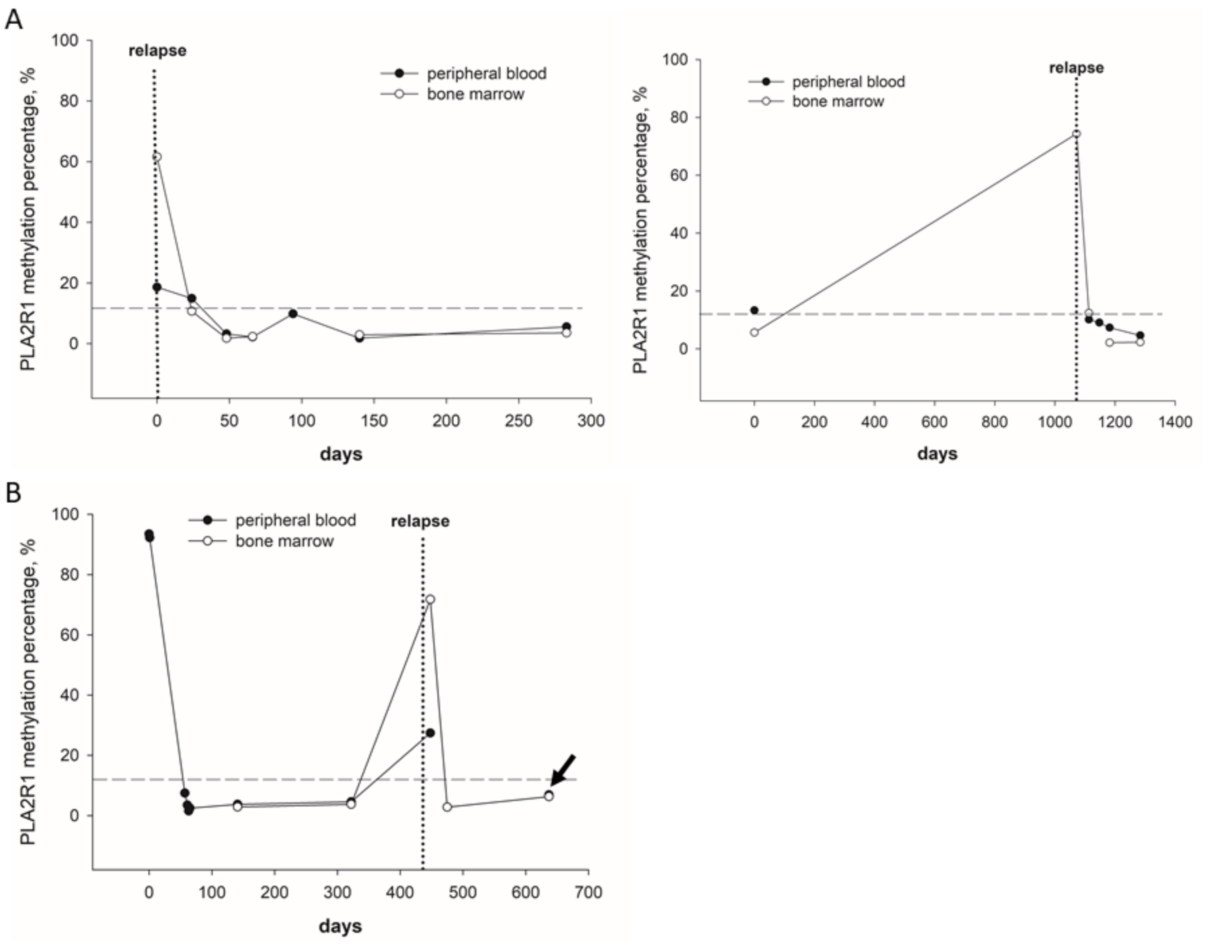

Supplementary Figure 3

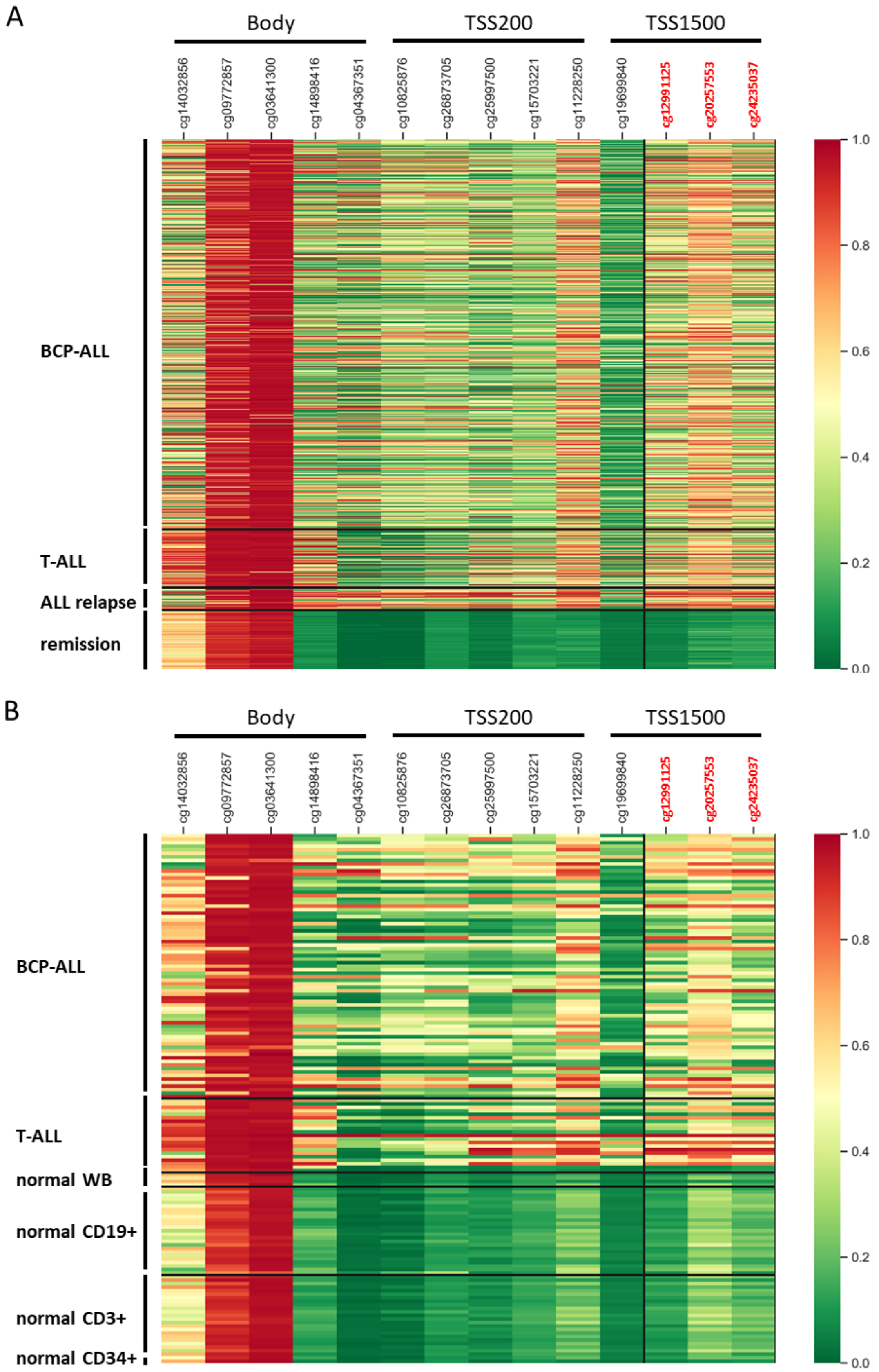

Supplementary Figure 4

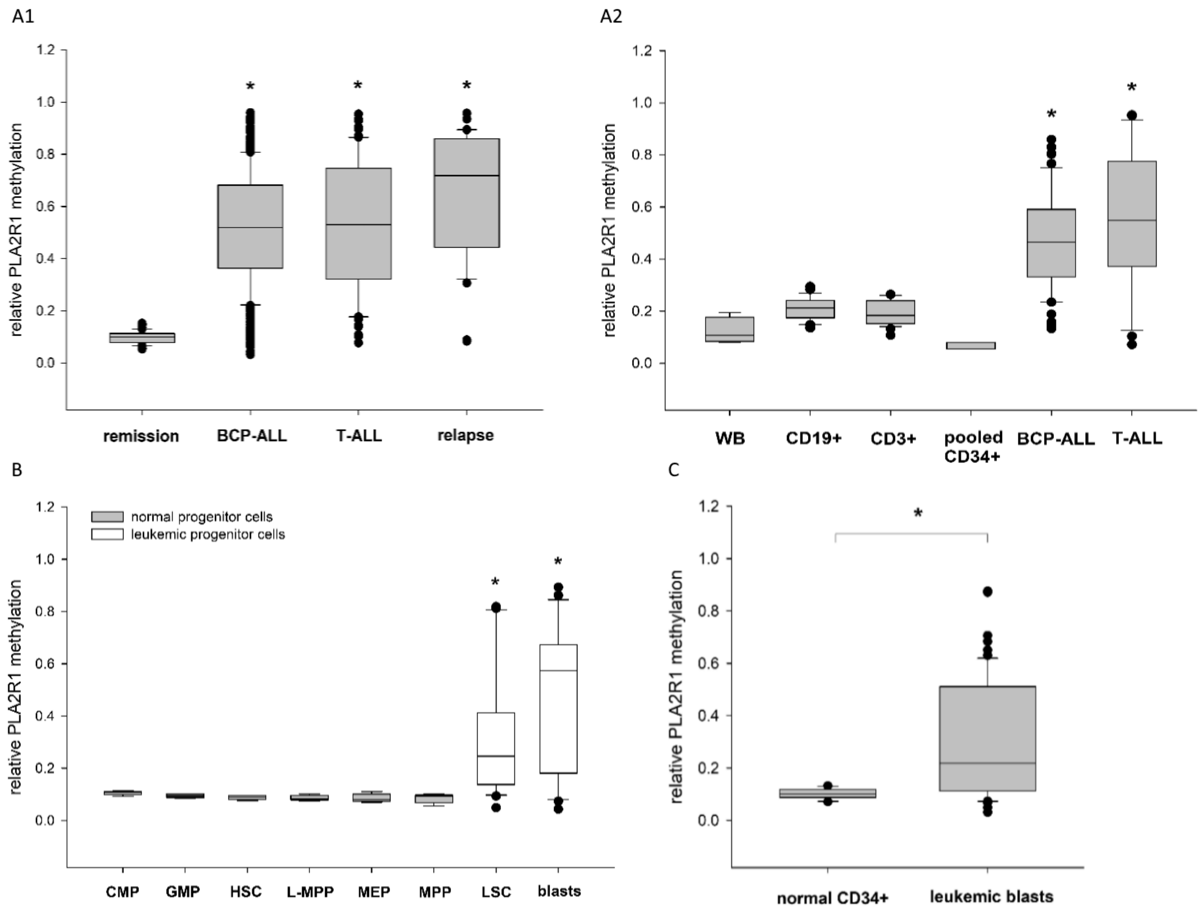

Supplementary Figure 5

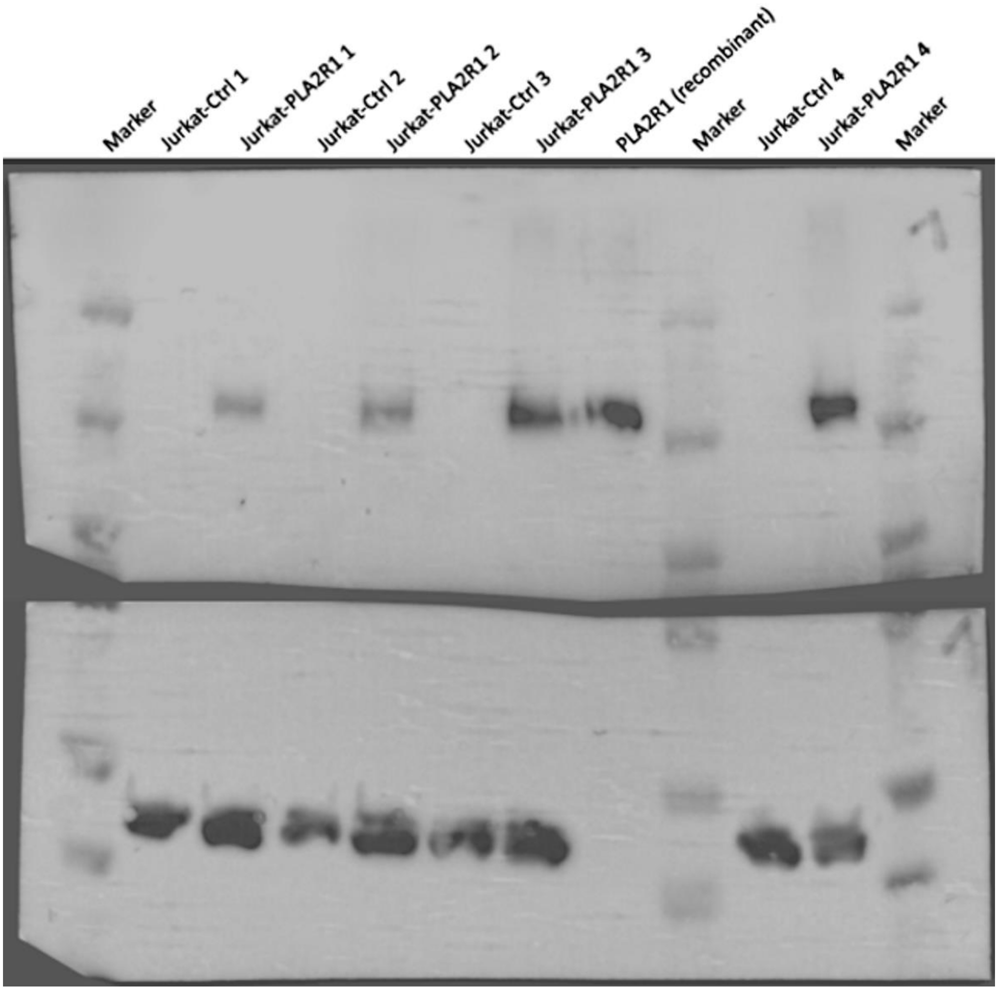

Supplementary Figure 6

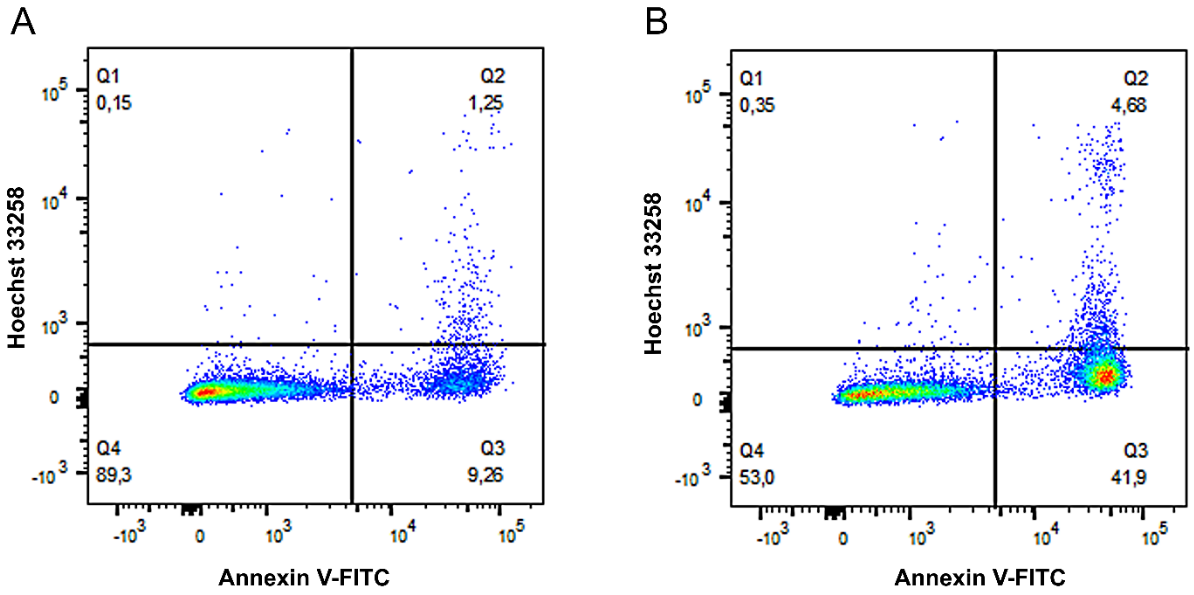

Supplementary Figure 7
